# The genomic and molecular landscape of splenic marginal zone lymphoma, biological and clinical implications

**DOI:** 10.37349/etat.2024.00253

**Published:** 2024-07-23

**Authors:** Amatta Mirandari, Helen Parker, Margaret Ashton-Key, Benjamin Stevens, Renata Walewska, Kostas Stamatopoulos, Dean Bryant, David G. Oscier, Jane Gibson, Jonathan C. Strefford

**Affiliations:** University of Bologna, Italy; ^1^Cancer Sciences, Faculty of Medicine, University of Southampton, SO16 6YD Southampton, UK; ^2^Department of Pathology, University Hospital Southampton NHS Foundation Trust, SO16 6YD Southampton, UK; ^3^Department of Molecular Pathology, University Hospitals Dorset, SO16 6YD Bournemouth, UK; ^4^Institute of Applied Biosciences, Centre for Research and Technology Hellas, 57001 Thessaloniki, Greece

**Keywords:** Lymphoma, genomics, haematology, therapeutic targets, splenic marginal zone lymphoma, biomarkers, clinical outcome

## Abstract

Splenic marginal zone lymphoma (SMZL) is a rare, predominantly indolent B-cell lymphoma constituting fewer than 2% of lymphoid neoplasms. However, around 30% of patients have a shorter survival despite currently available treatments and the prognosis is especially poor for the 5–15% of cases that transform to a large cell lymphoma. Mounting evidence suggests that the molecular pathogenesis of SMZL is critically shaped by microenvironmental triggering and cell-intrinsic aberrations. Immunogenetic investigations have revealed biases in the immunoglobulin gene repertoire, indicating a role of antigen selection. Furthermore, cytogenetic studies have identified recurrent chromosomal abnormalities such as deletion of the long arm of chromosome 7, though specific disease-associated genes remain elusive. Our knowledge of SMZL’s mutational landscape, based on a limited number of cases, has identified recurring mutations in *KLF2*, *NOTCH2*, and *TP53*, as well as genes clustering within vital B-cell differentiation pathways. These mutations can be clustered within patient subgroups with different patterns of chromosomal lesions, immunogenetic features, transcriptional signatures, immune microenvironments, and clinical outcomes. Regarding SMZL epigenetics, initial DNA methylation profiling has unveiled epigenetically distinct patient subgroups, including one characterized by elevated expression of Polycomb repressor complex 2 (PRC2) components. Furthermore, it has also demonstrated that patients with evidence of high historical cell division, inferred from methylation data, exhibit inferior treatment-free survival. This review provides an overview of our current understanding of SMZL’s molecular basis and its implications for patient outcomes. Additionally, it addresses existing knowledge gaps, proposes future research directions, and discusses how a comprehensive molecular understanding of the disease will lead to improved management and treatment choices for patients.

## Introduction

The term “splenic marginal zone lymphoma” (SMZL) was introduced by Schmid in 1992 to describe the splenic histology and immunophenotype in four patients who presented with splenomegaly, anaemia, and weight loss [1]. All patients showed expansion of the splenic marginal zone by B-cells with morphological and immunohistochemical features that were distinct from follicular lymphomas (FLs) or mantle cell lymphomas (MCLs). Like other marginal zone lymphomas (MZLs), including nodal MZL (NMZL) and extranodal MZL (EMZL), SMZL is associated with a history of prior chronic infections, auto-immune conditions, and nuclear factor kappa B (NF-κΒ) pathway dysfunction. There remains uncertainty as to whether all MZLs share a common cell of origin.

Recently, two separate classifications of haematolymphoid tumours have been published. The International Consensus Classification of Mature Lymphoid Neoplasms retains the definition of SMZL, like that in the 4th edition of the World Health Organization (WHO) classification [2], as a mature B-cell neoplasm primarily involving the spleen alongside hairy cell leukaemia (HCL), HCL variant (HCL-V), splenic diffuse red pulp lymphoma (SDRPL), and splenic B-cell lymphoma/leukaemia, unclassifiable. In contrast, the 5th edition of the WHO classification lists SMZL as one of four splenic leukaemia and lymphomas together with HCL, SDRPL, and a new category: splenic B-cell lymphoma/leukaemia with prominent nucleoli (SBLPN) [3]. The latter encompasses cases previously diagnosed as HCL-V, some cases of B-prolymphocytic leukaemia and a small subset of cases previously diagnosed as SMZL. It seems likely that further biological studies in this subset will determine whether SBLPN represents a distinct condition or variants of previously recognised disorders [4].

SMZL comprises about 2% of lymphoid malignancies and approximately 0.6% of non-Hodgkin lymphoma (NHL) cases, with an incidence rate of 0.13 per 100,000 individuals per year [5, 6]. The median age range of diagnosis is 65–70 years and epidemiological studies have shown no consistent male or female preponderance [7–9]. Diagnosis may occur following the incidental discovery of lymphocytosis or the detection of splenomegaly during examination or imaging. However, the typical presentation often involves abdominal fullness or pain due to significant splenomegaly, and/or symptoms associated with cytopenia [10, 11]. SMZL has been associated with autoimmune conditions [12], and there is a significant correlation between SMZL and hepatitis C virus infection in southern European populations and China [13, 14]. Lymphadenopathy, when present, is usually intra-abdominal (especially splenic hilar or intra-thoracic) and rarely involves peripheral nodes. Systemic symptoms such as fever and night sweats should raise the possibility of a high-grade transformation.

Whilst splenic histology provides a definitive diagnosis, splenectomy is now rarely performed for either diagnostic or therapeutic reasons and diagnosis is usually based on a combination of clinical findings, lymphocyte morphology, bone marrow histology, immunohistochemical and immunophenotypic features [15]. The typical immunophenotype includes surface expression of IgM, CD20, CD27, CD49d, and variable expression of IgD, CD5, CD11c, and CD25 [16]. Lack of expression of CD103, CD123, annexin A1, and cyclin D1 serve to distinguish SMZL from other splenic lymphomas (Table 1). Additionally, the CD200/CD180 median fluorescence (MFI) ratio may help to distinguish SDRPL diagnosis over HCL, SMZL, and SBLPN, where a ratio of 0.5 or less is in favour of SDRPL [3]. Cases presenting with isolated lymphocytosis, morphological and immunophenotypic features suggestive of SMZL, but of which lacks splenomegaly (as well as lymphadenopathy) on imaging fulfil the diagnostic features of clonal B-cell lymphocytosis of marginal zone origin (CBL-MZ). CBL-MZ has been included in the new WHO classification, as a non-chronic lymphocytic leukaemia (CLL)/small lymphocytic lymphoma (SLL)-type monoclonal B-cell lymphocytosis (MBL) entity, that frequently harbour features consistent with a MZ origin [3]. On follow-up, a minority of CBL-MZ cases progress to SMZL or another MZL [17].

**Table 1:** Similarity and differences in features of SMZL, HCL, and SDRPL

**Features**	**Type of feature**	**SMZL**	**HCL**	**SDRPL**
Demographics	Incidence	0.13/100,000	0.4/100,000	?
	Median age	65–69	55–63	70
	M:F ratio	1:1.1 to 1:1.85	3:1 to 4:1	1.6:1 to 2.4:1
Immunophenotype	CD25	+	++	-
	CD27	++	--	-
	CD5	+	-	-
	CD200	-	++	-
Outcome	Need for treatment	70%	Yes	50%
	Transformation	10–20% into DLBCL	5–6%	Rarely

+: indicates positive; ++: indicates strong positive; -: indicates negative; --: indicates strong negative; ?: unknown. SMZL: splenic marginal zone lymphoma; HCL: hairy cell leukaemia; DLBCL: diffuse large B-cell lymphoma; SDRPL: splenic diffuse red pulp lymphoma; M:F: male: female

SMZL frequently pursues an indolent course with a median survival of 10–15 years and only 40–50% of deaths are disease or treatment-related. Despite this generally favourable outcome, the prognosis of SMZL can be heterogenous, wherein 30% of cases have a shorter survival despite currently available treatments [18] and the prognosis is especially poor for the 5–15% of cases that undergo transformation to a large cell lymphoma [19–21]. Importantly, in comparison to other MZLs, SMZL patients have the greatest risk of transformation, which results in significantly decreased survival [22, 23]. The rarity and indolence of the disease have hindered the development of specific treatment options for SMZL, with most current treatment regimens only being supported by retrospective studies or based on retrospective series or trials involving broader indolent B-cell lymphoma subtypes [7].

**Figure 1:**
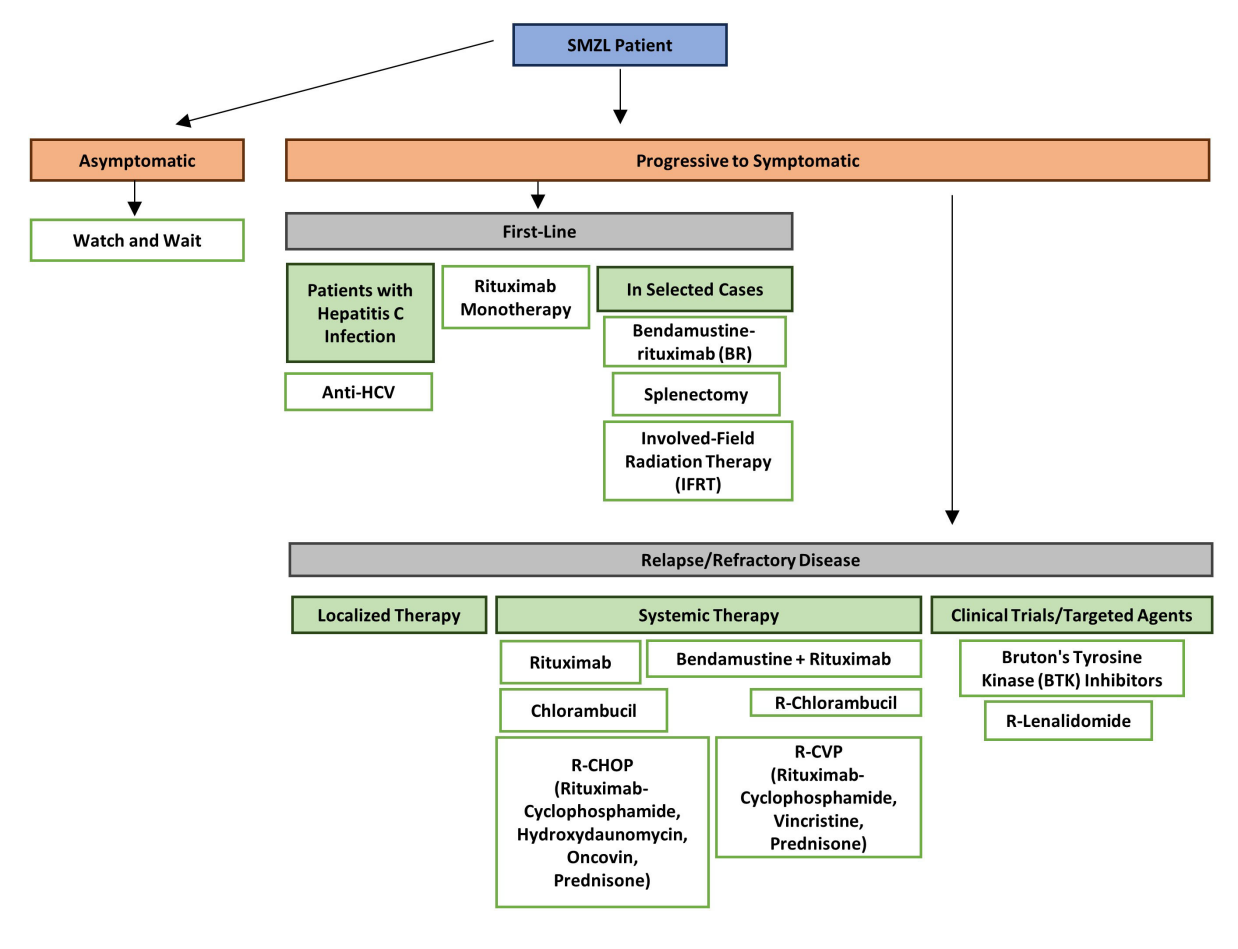
Flowchart of treatment guidelines for SMZL [24]. SMZL: splenic marginal zone lymphoma; HCV: hepatitis C virus

Indication to begin treatment should be based on evidence of symptomatic or progressive disease, as outlined by the latest guidance for the diagnosis and treatment of MZLs [18, 24] (Figure 1). An overview of the consensus guidance includes the following: (1) the minority of cases with hepatitis C infection should receive up-front anti-viral therapy; (2) for the majority of cases, rituximab monotherapy is the preferred initial therapy with high response rates and durable responses; (3) combination immune-chemotherapy such as rituximab + bendamustine is also highly effective, and can result in prolonged responses especially in cases who achieve minimal residual disease (MRD) negativity after 3 cycles of treatment [25] but in view of the high incidence of adverse events, is only an option for younger, fitter patients; (4) although long term outcome data for splenectomy is comparable to rituximab in many studies, especially when performed in experienced centres, the advantage of obtaining a precise diagnosis is offset by the risk of post-operative complications and requirement for infection prophylaxis. However, if splenectomy is undertaken, access to splenic tissue provides a unique opportunity to study tumour-microenvironmental interactions, let alone establish an unequivocal diagnosis; (5) patients who relapse after 2 years from initial rituximab treatment are candidates for re-treatment with the same regimen; (6) for those with early relapse or with refractory disease, Bruton’s tyrosine kinase (BTK) inhibitors, especially second-generation inhibitors zanubrutinib and acalabrutinib, have shown benefit in clinical trials. Given the activation of phosphoinositide 3-kinase (PI3K) signaling in a broad spectrum of B-cell tumours [26, 27], it is unsurprising that these inhibitors are also active in relapsed/refractory MZL, though they are not licensed for use due to their significant toxicity [24]. Lastly, as in other B-cell tumours, there is increasing interest in the use of immune therapies including immune checkpoint inhibitors, bispecific antibodies, and chimeric antigen receptor T-cell (CAR-T) therapy.

SMZL is associated with a number of molecular characteristics, including recurring genomic abnormalities which include del(7q) (approximately 40% cases), *KLF2* (20–30%), *NOTCH2* (10–25%), *TP53* mutations (10–15%), biased IGHV (immunoglobulin heavy variable) gene repertoire with usage of IGHV1-2*04 in 30% of cases, as well as key transcriptomic and epigenetic features of the tumour cells. As of now, there are yet to be any actionable and predictive biomarkers to stratify SMZL patients with a risk of undergoing a more aggressive clinical course. The biological and clinical implications of such genetic and molecular characteristics are discussed in detail in this review.

## Immunogenetics implicate antigenic drive in SMZL

SMZL development is shaped significantly by antigen selection and B-cell receptor (BCR) stimulation, demonstrated by the preferential usage of specific IGHV genes, particularly IGHV1-2 (30%), IGHV4-34 (11%), and IGHV3-23 (9%) [20, 21]. The IGHV1-2*04 allele is notably enriched in SMZL cases, representing 30% of all cases and 90% of IGHV1-2 users [28–30]. The usage of this allele is remarkable as it is much less frequent in other types of B-cell lymphomas [31] and is significantly associated with del(7q), *KLF2*, and *NOTCH2* mutations [28, 29, 32].

SMZL displays somatic hypermutation (SHM) in the great majority of cases [33]. Of note, most (approximately 75%) IGHV1-2*04 cases exhibit borderline mutated status (97–99% germline identity), displaying a precise pattern often targeting framework regions (FRs) [30]. Intra-clonal diversification with targeted ongoing SHM is also observed [34]. A minority of cases harbour 100% germline IGHV identity and exhibit reduced time to treatment [29].

The IGHV1-2*04 allele is particularly distinctive as it carries a tryptophan instead of an arginine at position R-75 of its FR3. This alteration is theorized to lead to structural changes that are important for the recognition of specific antigenic epitopes [30]. Specific IGHD genes (IGHD3-3 and IGHD3-10) and IGHJ genes, along with longer heavy complementarity determining region 3 (VH CDR3) sequences, are also observed. Recombinant monoclonal antibodies from IGHV1-2*04 SMZL demonstrated reactivity with various human cell antigens and human serum, indicating both poly- and self-reactivity [35]. Taken together, cases carrying the IGHV1-2*04 allele are believed to represent a distinct molecular variant of SMZL in the context of immune-driven initiation, leading to lymphoproliferation associated with autoimmunity and/or ongoing SHM [36].

## Molecular and genetic features of SMZL

### Common cytogenetic alterations

**Figure 2:**
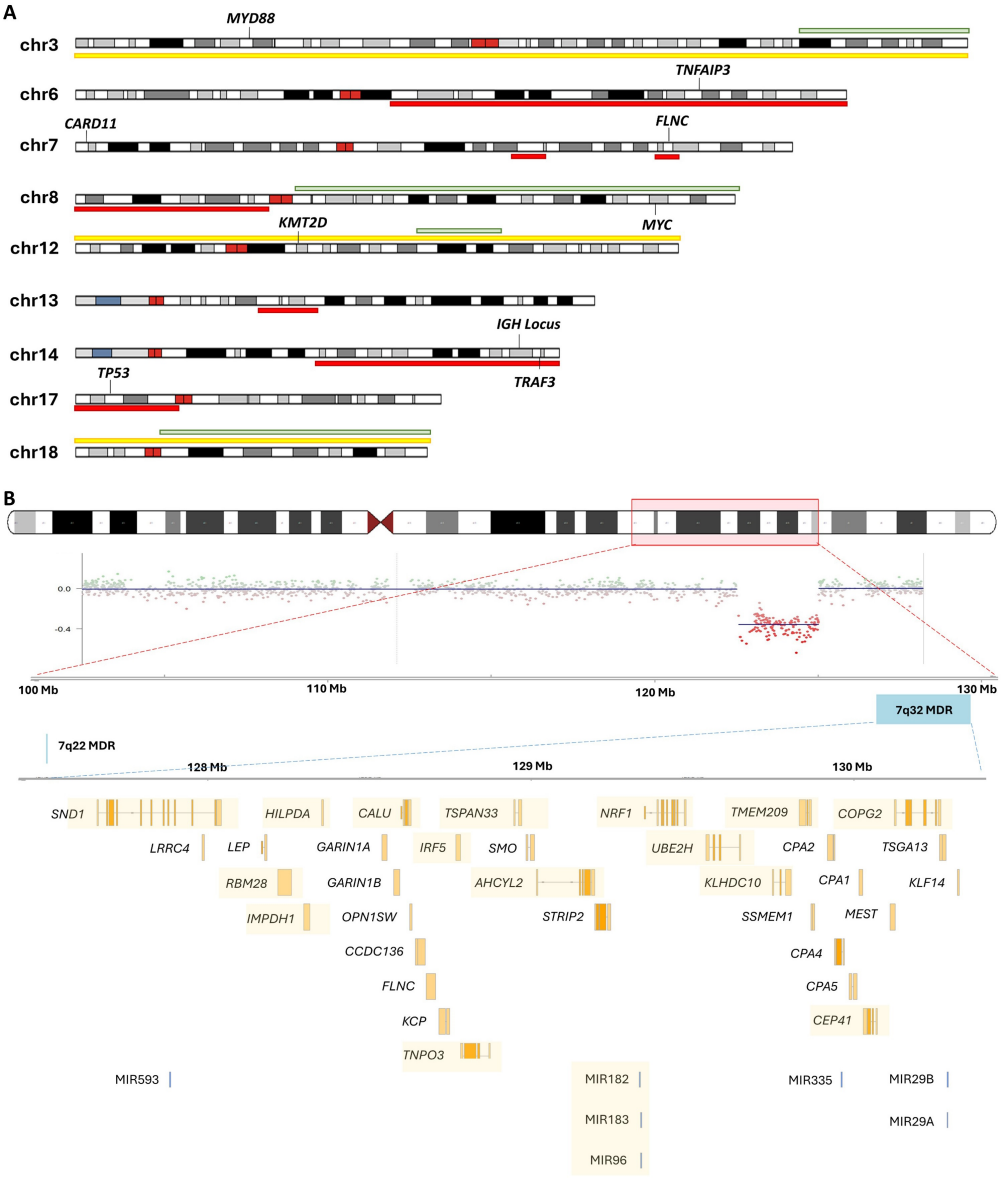
(A) Ideogram of recurrent copy number alterations in SMZL. Yellow indicates a trisomy, while red and green indicate common regions of deletions and gains, respectively; (B) schematic diagram of the recurrent 7q deletion in SMZL. The chromosome 7 ideogram indicates the common breakpoints of deletion found among SMZL cases. The panel below shows an example of a copy number plot of a case with clear lower segmentation means indicating the 7q deletion. A total of 38 coding genes and 6 microRNAs (miRNAs) are found in the 7q32 minimally deleted region (MDR)—those that were found to be transcriptionally under-expressed are indicated by the yellow highlight [43]. SMZL: splenic marginal zone lymphoma

Copy number alterations (CNAs) and karyotypic complexity are key features with clinical utility in other B-cell neoplasms such as CLL [37, 38]. In SMZL, a G-banding study of 330 cases found that 50% of cases harboured complex karyotypes (as defined by having ≥ 3 cytogenetic aberrations per metaphase cell), while 30% of cases harboured single aberrations, and 18% of cases harboured two [39]. The most common abnormalities include 7q22-q32 deletions and chromosome 3/3q gains (approximately 25%, Figure 2A, Table 2). Complex karyotypes involving chromosomes 1, 3, 7, 8, and 14 are also frequent [39–41]. *TP53* deletions, occurring within the del(17p) minimally deleted region (MDR), are found in 8–20% of SMZL cases and are associated with adverse outcomes [39, 42].

**Table 2:** Recurrent molecular lesions in SMZL

**Lesion**	**Prevalence**	**Genomic location, target genes, functions, and associations**	**References**
Copy number alterations			
del(7q)	14–44%	Under-expression of 18 genes and 8 miRNAs in the 7q32.1-32.2 MDR, rare mutations in the *FLNC* gene.	[29, 43–47]
dup(3q)	15–34%	Breakpoints typically span 3q26-q29, associated with gains in chromosome 12, common to several MZLs.	[39, 44]
+12	8–25%	+12 is associated with worse overall survival in univariate analysis, but no correlation with event-free survival.	[43]
+18	8–23%	Mutually exclusive in samples with a 3q gain.	[39]
del(17p)	8–24%	*TP53* gene. Faster clearance and elimination of rituximab.	[39, 42]
del(13p)	5–18%	Same as MDR in CLL. del(13q) presented a slower rate of elimination of rituximab.	[48]
del(14q)	3–10%	30–40% harbour translocations involving *BCL3*.	[49, 50]
del(6q)	8–24%	No candidate genes were found in a small series of transformed SMZL.	[51]
dup(8q)	2–20%	Associated with poor clinical outcomes if inclusive of the *MYC* gene locus.	[52]
del(8p)	4–15%	Associates with poor outcomes. Amongst MZLs, is only common in SMZLs.	[48]
Somatic mutations			
*NOTCH2*	10–25%	Crucial for MZ B-cells differentiation, associated with 7q deletions, *KLF2* somatic mutations, and IGHV1-2*04 usage, an independent risk factor for TTFT (multivariate analysis).	[29, 53–59]
*NOTCH1*	Approximately 5%	Mutations cause NOTCH1 activation in several mature B-cell tumours.	[60–62]
*SPEN*	Approximately 5%	Negative regulator of B lymphocyte differentiation into MZ B cells.	[63]
*TNFAIP3*	7–15%	Mutations resulted in a loss of NF-κB cascade inhibition in other NHLs.	[64]
*KLF2*	12–42%	Most frequent mutation in SMZL, *KLF2* knockout murine B-cells have impaired migration and MZ differentiation, through NF-κB deregulation.	[28, 29, 56, 59, 65–68]
*CARD11*	Approximately 5%	Involved in the BCR and NF-κB signaling pathways.	[54, 69]
*TRAF3*	Approximately 8%	TRAF3 inactivation leads to B-cell lymphomagenesis in mice, through a non-canonical NF-κB pathway.	[70]
*TP53*	Approximately 15%	Key tumour-suppressor that promotes cell-cycle arrest and apoptosis. Often deleted. Associated with unfavourable overall survival.	[29, 52, 71]
*MYD88*	5–15%	A small proportion of *MYD88* mutations in SMZL are L265P (6%). Role in MZ B-cell differentiation, and its absence leads to a deficiency in their availability. Promotion of B-cell proliferation and survival by activation of NF-κB.	[72–79]
*KMT2D*	11–15%	Frequent in all lymphoid tumours, mutants have delayed GC formation.	[80, 81]
*CREBBP*	Approximately 5%	Mutations were found to be fully clonal and likely early initiating events, like that in FL. Frequent in DLBCL and FL. Regulates enhancer networks with a crucial role in GC/post-GC cell fate decisions.	[53, 82, 83]

BCR: B-cell receptor; SMZL: splenic marginal zone lymphoma; MZLs: marginal zone lymphomas; MDR: minimally deleted region; CLL: chronic lymphocytic leukaemia; MZ: marginal zone; NF-κB: nuclear factor kappaB; GC: gastric cancer; FL: follicular lymphoma

In SMZL, the most prominent cytogenetic feature is del(7q), detected in 14–44% of patients [39, 44, 45, 48]. The breakpoints have been shown to vary from 7q21 to q36, but a 3.04 Mb MDR at 7q32.1-32.2 (127.03–130.07 Mb) has been identified, with another less common deletion region at 7q22 [43, 45, 46] (Figure 2B). A study comparing cases with or without del(7q) identified 38 coding genes within the 7q MDR [43, 46], and of those genes, 18 were significantly under-expressed in cases with the deletion and some of these had further evidence for hypermethylation [46]. Additionally, a cluster of six miRNAs in the del(7q) MDR exhibited reduced expression, including miR-593, miR-129, miR-182, miR-96, miR-183, miR-335, miR-29a, and miR-29b1 [46]. Integrated CNA and whole-exome sequencing (WES) analysis identified three mutated putative candidate genes of this deletion, *CUL1*, *EZH2*, and *FLNC*, with *FLNC* located within the mapped MDR [53].

The second most common aberration is the dup(3q), which is present in up to 34% of patients and is sometimes the result of unbalanced translocations [44, 48]. In contrast to other MZLs, where whole trisomy 3 is predominant, SMZL frequently exhibits a gain in 3q23, often in conjunction with a complex karyotype [39]. The breakpoints and partners in dup(3q) rearrangements vary, indicating secondary events [84]. Compared with the more aggressive diffuse large B-cell lymphoma (DLBCL), SMZL genomes infrequently harbour balanced chromosomal translocations, though IgH locus translocations can occur (9% of cases) in t(14;19)(q32;q13), t(6;14)(p21;q32), t(9;14)(p13;q32), and t(1;14)(q21;q32), targeting *BCL3*, *CCND3*, *PAX5*, and *BCL9*, respectively [39, 85]. The (14;19)(q32;q13) translocation was first discovered in CLL, with the identification of *BCL3* as the translocation partner of the IgH locus [49]. A cluster of breakpoints in the 3’ region of *BCL3* was shown to be associated with cases of atypical SMZLs with mutated IGHV status and complex karyotypes-this breakpoint did not, however, result in changes in expression of the *BCL3* [86]. Hence, in such cases, it is still not clear what the relevant target genes of these translocations may be [50].

### SMZL mutations cluster within common pathways

Genome-wide studies, employing unpaired whole-genome sequencing (WGS), WES, and targeted sequencing technologies, have revealed recurrently mutated genes and pathways in SMZL development. However, these approaches have limitations in exploring non-coding genome regions and in investigating mutational signatures contributing to disease. *KLF2* (12–42%), *NOTCH2* (10–25%), and *TP53* (15%) are among the most frequently mutated genes [28, 32, 53–55] (Table 2). Recurrent truncating mutations are observed in *TNFAIP3*, *KMT2D*, and *MYD88*, where the latter results in the promotion of B-cell proliferation and survival by activation of NF-κB [72]. Additionally, other genes within pathways that relate to crucial B-cell functions are also mutated, including the NOTCH pathway, NF-κB signaling, chromatin remodeling, cytoskeleton organization, and toll-like receptor (TLR) signaling pathways.

#### The NF-κB pathway is deregulated in SMZL

In B-cells, NF-κB activation influences signaling pathways and downstream genes fundamental to development, differentiation, and survival [87, 88]. NF-κB is typically sequestered in an inactive state in the cytoplasm by inhibitory IκB proteins. There are two pathways of NF-κB signaling: canonical and non-canonical. These pathways involve distinct NF-κB family members and are triggered by specific receptor-proximal signals, such as those emanating from the BCR, tumour necrosis factor-alpha (TNF-α), TLRs, and interleukin-1 (IL-1). It involves phosphorylation and degradation of IκB proteins by IκB kinase α (IKKα) and IKKβ, releasing and activating NF-κB heterodimers (e.g., p65/RelA) that translocate to the nucleus to regulate gene expression. Conversely, the non-canonical pathway is activated by stimuli like B-cell activating factor (BAFF) and a proliferation-inducing ligand (APRIL), which bind to receptors such as BAFF-receptor (BAFF-R) and CD40, leading to activation of IKKα. This results in the processing of p100 to p52-REL-B heterodimers, which regulate gene expression. The TNFAIP3 (A20) protein and other negative feedback mechanisms modulate its activation [89].

**Figure 3:**
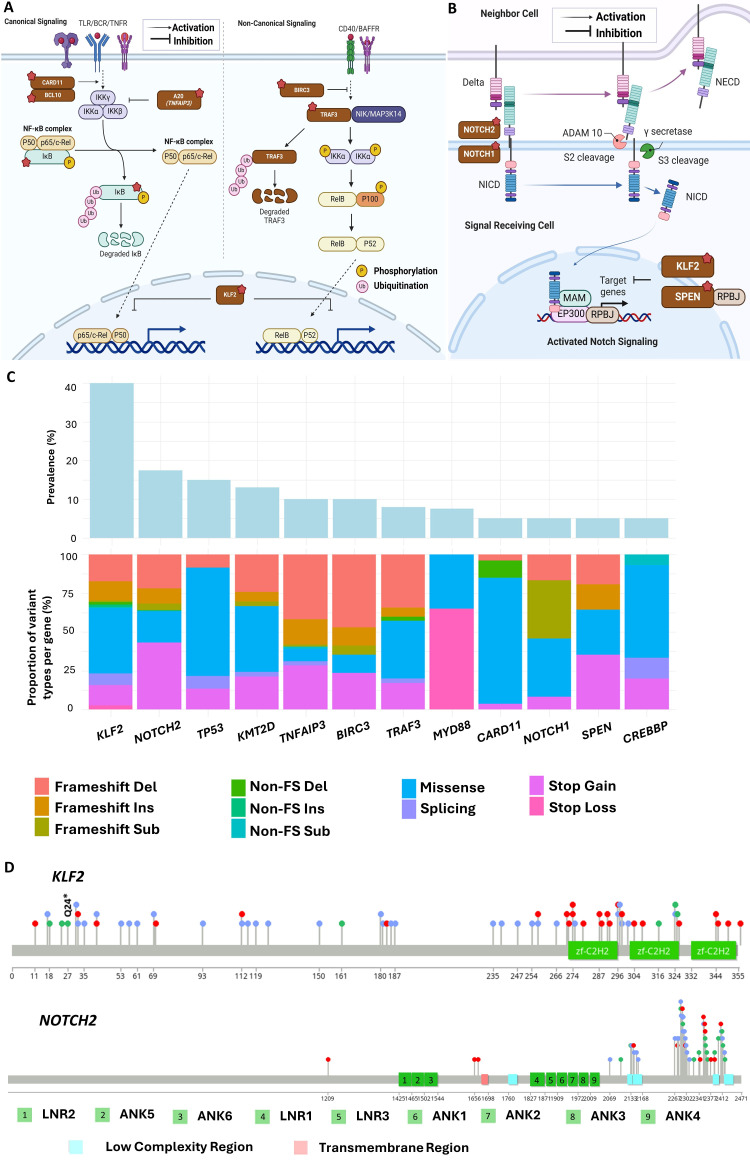
Recurrently mutated genes and pathways in splenic marginal zone lymphoma (SMZL). (A) Illustration of the NF-κB signaling pathway. Response to diverse stimuli, including ligands of B-cell receptors (BCRs) and toll-like receptors (TLRs) leads to the primary mechanism of nuclear factor kappa B (NF-κΒ) activation through the inducible degradation of the IκB, which is triggered by the site-specific phosphorylation of the multi-subunit IκB kinase (IKK) complex. Recurrent coding mutations in *KLF2*, *TRAF3*, *TNFAIP3*, *BIRC3*, *CARD11*, and *BCL10* are recurrently found across SMZL; (B) illustration of the NOTCH signaling pathway. When NOTCH2 is activated by the binding of ligands such as Jagged ligands and delta-like ligands, its intercellular domain [Notch intracellular domain (NICD)] is cleaved and released into the cytosol, where it then traffics into the nucleus to act as a transcriptional regulator. Mutations in *SPEN*, *NOTCH1*, and *NOTCH2* are recurrently found across SMZL; (C) types of unique mutations found across recurrently aberrant genes in SMZL; (D) distribution of mutations found across *KLF2* (top) and *NOTCH2* (bottom) in SMZL. TNFR: tumour necrosis factor receptor; BAFFR: B-cell activating factor receptor; NICD: Notch intracellular domain; NECD: Notch receptor extracellular domain; MAM: mastermind; TRAF3: tumour necrosis factor-receptor associated factor; FS: frameshift; RPBJ: recombination signal binding protein for immunoglobulin kappa J region. Figure 3A and 3B were created with Biorender.com *Note.* Figure 3A was adapted from “Notch Signaling Pathway”, by BioRender.com (2020). Retrieved from: https://app.biorender.com/biorender-templates/figures/all/t-5f6d097dbedf1200aa63bc9c-notch-signaling-pathway; “NF-KB Signaling Pathway”, by Iwasaki A (2020). Retrieved from: https://app.biorender.com/biorender-templates/figures/all/t-5f5b7c3514d40300aa942984-nf-kb-signaling-pathway

Constitutive NF-κB activation has been implicated in the growth of various B-cell leukaemia and lymphomas, with mutations in multiple genes playing a part. Constant NF-κB activation in MZ B-cells models results in abnormal B-cell expansion [90], and enhanced signaling is observed in other lymphoid malignancies including Hodgkin lymphomas, DLBCL, and multiple myeloma [91]. Genetic abnormalities in genes involved in this pathway including *CARD11*, *TNFAIP3*, *TRAF3*, and *BIRC3* [53, 54, 92] have also previously been reported (Figure 3A).

The TNF-receptor associated factor (TRAF) proteins act as docking molecules for the interaction between the cytosolic regions of various tumour necrosis factor receptors (TNFRs) and TLRs. Studies have shown the action of the A20 protein, encoded by the *TNFAIP3* gene, inhibits NF-κΒ activation in the TNFR1 signaling pathway with the help of several binding partners including TRAF1 and TRAF2 [93, 94]. *TRAF3* has recurrently mutated in a variety of B-cell malignancies, including SMZL (21%) [95]. Inactivation of *TRAF3* has been found to increase the survival of mature B-cells, leading to the development of B-cell lymphomas in murine models [70]. Mutational clusters were found within the C-terminal domain of the protein, which is required for BIRC3 protein recruitment. Alternatively, truncating mutations within the RING domain of BIRC3 occur in 10% of SMZLs and result in the loss of ubiquitin ligase activity, further stabilizing NF-κB-inducing kinase (NIK) and resulting in the constitutive upregulation of NF-κB targets [93, 96]. Mutations that result in the activation of positive regulators of the NF-κB pathway, such as in *CARD11* and *IKBKB*, have also been identified in a proportion of SMZL cases [93, 97].

#### TLR signaling acts as the critical activator of the NF-κB pathway

The TLR pathway plays a significant role in the development of SMZL by acting as a critical activator of the NF-κB pathway itself [98]. Of particular importance is the MyD88 adaptor protein, which is essential for transmitting signals through the TLRs (other than TLR3) as well as the IL-1 receptor family. The *MYD88* gene is found to be mutated in up to 15% of SMZL cases [73, 69]. Functional studies have demonstrated that MyD88 is necessary for MZ B-cell differentiation [74]. Common mutations within the Toll/interlukin-1 receptor (TIR) domain common to TLR and IL-1 family of receptors result in structural changes resulting in MyD88-dependent recruitment of IRAK-1/4 and enhanced NF-κB signaling [99]. The L265P mutation has been observed in various lymphoid malignancies, particularly including lymphoplasmacytic lymphoma (LPL) [73, 75]. This gain-of-function alteration has also been found in SMZL, associated with improved disease survival [76–78].

#### KLF2 is the most common somatic lesion in SMZL

*KLF2* emerges as the most recurrent mutation in SMZL, present in 12–40% of cases and strongly associated with usage of the IGHV1-2*04 allele and del(7q). KLF2 acts as a transcription factor, regulating key genes, including those in the NF-κB and NOTCH signaling pathways [65, 100] (Figure 3A and 3B). Its DNA binding and transcriptional control play vital roles in the differentiation and maintenance of mature peripheral B-cells, acting as a quiescence factor [66, 67]. In vivo studies show that KLF2 deficiency results in marked MZ B-cell expansion in murine models [68]. Impaired KLF2 also affects the migration of germinal cells to splenic MZ B-cells, influencing the trafficking and differentiation of MZ B-cells [66, 101].

Most *KLF2* mutations occur in the N-terminus and middle inhibitory domain, with missense mutations clustering in the zinc-finger 1 (ZF1) domain, impairing protein function [28] (Figure 3C and 3D). Truncating mutations disrupt the entire protein or remove ZF domains, including putative nuclear localization signal sequences [32, 56]. The recurrent nonsense mutation Q24* suggests a potential hotspot [29]. In vitro SMZL studies demonstrate that *KLF2* mutants result in significant loss of suppression in the NF-κB pathway, impacting various activating signaling pathways, such as those associated with the BCR, TLR, TNFR, and BAFF-R [28].

#### The NOTCH pathway is also important to SMZL development

*NOTCH2* has an important role in MZ B-cell development, differentiation, and homing [57, 58, 102]. It is a cell-surface receptor important in determining cell fate, proliferation, differentiation, and apoptosis [103] (Figure 3B). In MZ B-cells, conditional *NOTCH2* knockout resulted in defects in MZ B-cells and their presumed precursors of type 2 transitional B-cells [58]. Whilst common in SMZL, *NOTCH2* mutations are rare in CLL, MCL, and FL [60, 104]. In SMZL, frameshift indels and nonsense mutations predominate and cluster near the C-terminal PEST (proline, glutamic acid, serine, threonine)-rich domain [105] (Figure 3C and 3D). A recurrent p.R2400* nonsense mutation within this domain represents approximately 24% of *NOTCH2* mutations in SMZL [54]. Mutations in *NOTCH2*, like those targeting *KLF2*, are enriched in cases with 7q deletion and IGHV1-2*04 usage and are associated with adverse clinical outcomes [29, 55]. Immunohistochemical analysis using antibodies that recognize active nuclear NOTCH2 in situ supports *NOTCH2* activation in SMZL, but shows an expression of *NICD2* in both wild-type and NOTCH2 mutant tumours [106]. This suggests that akin to *NOTCH1* mutations in CLL, *NOTCH2* mutations in SMZL are secondary events that stabilize prior ligand-dependent Notch activation through selective pressure on this signaling pathway.

#### Mutations of TP53 are associated with poor outcomes in SMZL

Whilst *TP53* mutations are present in up to 15% of SMZL cases, concomitant with del(17p) in most cases, other genes are also recurrently mutation that are implicated in cell cycle control, such as *CCND3* (5%) and *ATM* (4%) [29, 107]. In SMZL, *TP53* disruptions primarily occur via missense mutations within its DNA binding domain, compromising its tumour suppressor function. Mutant p53 proteins lose the ability to activate canonical p53 target genes, leading to unchecked cell proliferation and the accumulation of genomic mutations, ultimately fostering tumour growth. *TP53* mutations signify a poor prognosis and are more prevalent in cases with unmutated IGHV genes, showing acquisition of del(17p) in a handful of transformed cases [29, 39, 48, 108].

#### Chromatin remodeling machinery is targeted by mutations in SMZL

Several genes involved in chromatin remodeling are frequently mutated in SMZL, highlighting the significance of epigenetic processes in disease initiation and development. These genes include *KMT2D* (11–15%), *ARID1A* (4–6%), *EP300* (2%), and *CREBBP* (5%) [29, 54, 109]. It should be noted that these gene mutations are found across several mature B-cell tumours, so have limited utility for differential diagnosis. KMT2D is a histone lysine methyltransferase that modifies lysine-4 of histone 3 (H3K4) marker, where mutant proteins have a functional role in DLBCL and FL [80]. The protein genacts as a bona fide tumour suppressor, and loss of its function has been shown to result in delayed germinal centre formation and overall promotion of lymphoma development in mouse models [81]. In SMZL, *CREBBP* mutations are clonal and appear to be early initiating events, like those found in FL [29]. The recurrent hotspot variant Y1450C has been shown to compromise its acetylation ability on Bcl-6 and p53 in DLBCL. *ARID1A* is crucial in maintaining chromatin accessibility at enhancer regions, and recurrent mutations have been observed in various cancer types [110, 111].

## Molecular classification systems in SMZL

In SMZL, several molecular classification systems have been used to help identify poor-risk patients. However, despite these advancements, further validation and investigations are needed to integrate these findings into clinical practice effectively. Overall refinement of SMZL sub-classifications and their clinical utility could conceivably help tailor treatments and improve outcomes for patients. In this section, we discuss some of the useful molecular sub-types that have emerged from recent research and how they contribute to our understanding of SMZL biology.

### Genomic subtypes

Bonfiglio et al. [59] identified subgroups of SMZL based on the enrichment of particular gene mutations clustering into specific biological pathways. The authors studied 303 splenic-derived tumour specimens from an international multicentre study (IELSG46) using a multi-omic approach. This study used a pathology expert panel, confirming the WHO classification for all cases. Using genetic and phenotypic disease features, they were able to identify a number of genetic subtypes, with differing survival outcomes. Most importantly, of the four total clusters, two prominent subgroups were proposed, termed NNK (mutations in NF-κB, *NOTCH*, and *KLF2*, 58.2% of all cases), and DMT [with DNA-damage response, mitogen-activated protein kinases (MAPK), and TLR modules, 32% of cases]. These sub-types exhibited different patterns of chromosomal lesions, immunogenetic features, transcriptional signatures, immune microenvironments, and clinical outcomes, with inferior survival in the NNK group.

The co-occurrence of mutations within the NNK cluster, which accounts for the majority of cases, alludes to a possible role of *KLF2* as a master regulator of both NOTCH and NF-κB signaling, suggesting the possible cooperation between these lesions in dysregulating MZ B-cell differentiation. As *KLF2*, an important transcription factor, is mutated in the immune-suppressive NNK SMZL subgroup, future studies might focus on how deregulation of KLF2 or downstream targets results in immune cell reprogramming, recruitment, or evasion, as has been shown for *CREBBP* and *EZH2* in other B-cell lymphomas [82].

#### DNA methylation subtypes

Recent studies have shown that epigenetic factors, such as changes in DNA methylation and histone modifications can play a critical role in establishing B-cell identify [112, 113]. In SMZL tumours, DNA methylation can contribute to the disruption of cell survival and proliferation pathways [114]. Arribas et al. [115] demonstrated that promotor DNA methylation patterns can be used to identify two distinct epi-clusters, based on the level of cytosine-guanine (CpG) methylation. They identified a high genome-wide promoter methylation subgroup (High-M) in 21% of their cohort, that displayed inferior overall survival, high risk of histologic transformation, and was enriched for IGHV1-2*04 usage, *NOTCH2* mutations, and del(7q). Gene promoters showing elevated methylation in the High-M group including PRC2 target genes, those harbouring tri-methylation marks (H3K27me3 and H3K4me3), those involved in chromatin remodeling, and a variety of tumour suppressor genes (including *KLF4*, *DAPK1*, *CDKN2A*, *WT1*, and *GATA4*). Hypomethylated promotors included those in PRC2 component genes, and those involved in cell cycle control and proliferation [115]. This study supports the presence of an aggressive tumour sub-group carrying established poor-risk molecular features with fundamental deregulation of EZH2 and broader PRC2 perturbation.

The global impact of DNA methylation can extend beyond the regulation of gene expression, and it has recently been shown that hypo-methylation in low CpG-content heterochromatin and hyper-methylation in high CpG-content polycomb regions is concomitant with ongoing cell division in B-cell tumours. This measure, termed epigenetically determined cumulative mitoses (epiCMIT) reflects the mitotic history of B cell tumours, both prior and subsequent to malignant transformation [116]. In addition to the initial paper which showed that epiCMIT scores can predict more aggressive disease in CLL, MCL, and DLBCL, there is recently emerging evidence for its relevance in SMZL as well. Parker et al. [117] profiled 142 SMZL patients drawn from international leaders in the management of the disease, using a variety of molecular approaches including DNA methylation analysis. In doing so, the authors made the following important observations; (1) that epiCMIT scores are highly heterogeneous in SMZL, suggesting that the quantity of tumour cell proliferation is highly variable between patients; (2) that epiCMIT scores negatively correlate with telomere length, supporting the association between cellular growth and telomere attrition; (3) that patients with high epiCMIT were more likely to carry other poor-risk features, such as IGHV1-2*04 usage, del(7q), mutations in key driver genes (*KLF2* and *NOTCH2*), and be classified in the High-M or NNK disease subgroups; and (4) that survival analysis selected epiCMIT as a strong independent marker of treatment free survival in univariate and multivariate models that account for other established clinical and molecular classification systems.

#### Transcriptional subtypes

Genome-wide transcriptional analysis of SMZL highlights the deregulation of genes within the BCR, TNFR signaling pathways, and those associated with NF-κB activation [118]. One specific example is the link between elevated *NOTCH2* expression and a more aggressive clinical course [119]. Bonfiglio et al. [59], in addition to their aforementioned genomic analysis, also performed transcriptional analysis and identified elevated expression of NOTCH pathway genes in the NNK genetic sub-class, supporting deregulation of non-canonical NF-κB transcription factors in this subtype. Conversely, the DMT-SMZL sub-class exhibited impaired p53 and apoptotic functions [59]. Other expression studies have implicated the deregulation of signal transduction, immunity, metabolic, and TLR pathways [82]. From the perspective of differential diagnosis, RNA analysis of a spectrum of B-cell lymphomas has identified a 135-gene SMZL-specific gene expression signature (SSGES) [120], including three specific genes that appear to be particularly important for distinguishing SMZL from other small cell lymphomas (*ILF1*, *SETX*, and *CD40*) [118]. In the High-M epigenetic cluster defined by Arribas and co-workers [115], the authors showed that several tumour suppressor genes were downregulated due to promoter methylation deregulation, including *KLF4*, *DAPK1*, *CDKN1C*, and *CDH1/2*. The authors propose that these genes might offer a survival advantage to tumour cells, with the potential of therapeutic targeting [115].

#### Non-coding RNAs in SMZL: preliminary insights from micro-RNA profiling

miRNAs are a subclass of non-coding RNA molecules varying in length, that play critical roles in gene regulation. At the genomic level, miRNA loci are often located within introns, frequently within clusters of multiple miRNAs that are expressed as a result of host gene transcription. miRNAs have been broadly implicated in the pathogenesis of other mature B-cell malignancies, with perhaps the most notable example being the analysis of del(13q14) in CLL, that down-regulated miR15a and miRNA16-1, negative regulators of MCL-1 and BCL2 [121, 122]. Again, data in SMZL is limited, but several miRNAs have been found to be dysregulated, including the down-regulation of miR-29a and miR-29b-1 [123]. A study comparing miRNA profiles of SMZL to reactive spleens revealed differential expression of 30 miRNAs, with nine of them also distinguishing SMZL from other B-cell lymphomas [124]. The hepatitis C virus has also been shown to downregulate miR-26b in SMZL [125]. Down-regulation of miR-593, miR-129, miR-182, miR-96, miR-183, miR-335, miR-29b1, and miR-29a is seen in 7q deleted patients, with *TCL1A*, *BCL2*, *RB1*, and *BCL-w* as a putative target genes [126, 127].

## Clinical implications of molecular features

Although molecular studies have provided insights into the biology of SMZL they are yet to be incorporated into routine practice. This reflects, in part, the rarity of the disease and paucity of clinical trials, the difficulty in making a definitive diagnosis and accessing suitable samples in some cases, and the limited availability of appropriate investigations. However, advances have been made to bridge current molecular understanding to emergent clinical hypotheses. This section aims to summarize the current understanding of the molecular landscape of SMZL, draw comparisons with other B-cell tumours, and discuss the implications for patient management and care in clinical settings.

### Diagnosis

No single genomic or immunogenetic marker is pathognomonic for SMZL. However, in the context of the differential diagnosis of a primary splenic lymphoma, the use of IGHV1-2*04 and/or del(7q) are very strong pointers for the diagnosis of SMZL, while a combination of these markers with or without mutations of genes within the NF-κΒ pathway is not seen in other splenic lymphomas. As discussed earlier in this review, transcriptomic studies may also have diagnostic potential. DNA methylation studies of normal B-cell maturation and more common B-cell tumours have identified both disease-specific signatures and differing patterns within diseases that have clinical implications. A recent study, published as an abstract, investigated DNA methylation profiles in splenic lymphomas with the pathological diagnoses of HCL, HCL-V, SDRPL, or SMZL, and identified 5 distinct methylation subgroups [128]. While the majority of the 170 SMZL cases formed a distinct subgroup, 29 were included in subgroups encompassing the new WHO classification of SBLPN. These cases were depleted in *NOTCH2*, *KLF2* mutations, and IGHV1-2*04 usage, suggesting either that they could be reassigned to the new WHO subgroup or there were differences in the criteria used to diagnose SMZL.

### Prognosis

Early studies indicated that both cytogenetic abnormalities and genomic mutations may have prognostic significance although the results were not always concordant among studies [54–56]. A cytogenetic study identified karyotypic complexity, 14q abnormalities and del(17p) as markers of a shorter overall survival in univariate, but not in multivariate analysis which included standard clinical and laboratory parameters and IGHV mutational status [39]. A multivariate analysis of 175 patients showed that *NOTCH2* mutations and 100% IGHV identity were associated with a shorter time to first treatment, while *TP53* mutations were associated with shorter overall survival [29]. It was, however, unclear whether *TP53* abnormalities impacted the survival of most patients with SMZL who received treatments that act through p53-independent mechanisms.

As lymphoma-specific deaths account for less than one half of total deaths in SMZL, Bonfiglio et al. [59] used a classifier and survival relative to a matched general population to assess excess mortality in patient subgroups based on genomics. After adjusting for the effects of age, sex, and year of diagnosis, the 10-year life expectancies of patients with NNK-, DMT-, and “immune-suppressive”-SMZLs were reduced by 21.0%, 14.5%, and 20.4% respectively. Cases with both the NNK genotype and an “immune-suppressive” microenvironment, had a relative survival of 70.8% at 10 years. Furthermore, 99.4% of mutations detected in the spleen were also identified in blood or marrow samples, offering less invasive avenues for biopsy procedures.

Additionally, epigenetic alterations have emerged as valuable clinical indicators, serving either as a metric for cellular proliferation [117], or as a global measure of hypermethylation (High-M) [115]. Both these studies emphasise how global changes in the epigenome, such as those correlated with cell proliferation, may have greater prognostic significance than any individual genomic abnormality. A recent study by Hopper and colleagues [129] employed bulk RNA-sequencing on a cohort of indolent lymphomas, including 48 SMZL tumours. They identified five putative patient clusters, three of which were strongly enriched for an SMZL diagnosis. One cluster, which the authors termed LGBCL5, was enriched for genes involved in cell cycle control, T-follicular helper cells, mutations in *TNFAIP3* and *BCL2*, and M1 macrophages in the tumour microenvironment (TME) inferred from the CIBERSORTx software. This cluster was also associated with a higher incidence of transformation to a high-grade lymphoma [129].

### Transformation

Histological transformation to a DLBCL is associated with a poor outcome in SMZL. In murine models, concurrent inactivating mutations of BLIMP1 and members of the NF-κB pathway can promote lymphomagenesis, leading to DLBCL with similar characteristics to human disease [130]. Genes involved in a proliferation signature were also found to be some of the most overly expressed upon the transformation of FL into DLBCL in human samples [131]. A study of 36 cases identified karyotypic complexity as the only risk factor for transformation in a multivariate analysis [21]. A more recent study [51] employed copy number arrays and a next-generation sequencing (NGS) panel to investigate genomic abnormalities in 41 SMZL patients with high-grade transformation, of whom 18 were studied at both diagnosis and at transformation (SMZL-T). The median time from diagnosis to transformation was 2.4 years (range 0–17) and the median survival from transformation was 6.55 years. SMZL-T clones arose by divergent evolution from a common altered precursor cell. Compared to SMZL, SMZL-T had higher genomic complexity. The most frequent abnormalities in SMZL-T were *TNFAIP3* (59.4%), *KMT2D* (46.9%), *TP53* (34.4%), *ARID1A* (31.3%), and *KLF2* (31.3%) mutations, losses of 9p21.3 (*CDKN2A/B*, 40.6%) and 7q31-q32 (34.4%), and gains of 1q (40.7%). Four cases with a *TP53* mutation at diagnosis, acquired a del(17p) at transformation. The main genomic variables predicting shorter survival from transformation were *KLF2* mutations, a complex karyotype, and a trend for *MYC* gains. These results identify a subgroup of patients in whom more regular follow-up could be indicated, although it is unclear whether earlier treatment would improve outcomes.

## Future perspectives and conclusion

Despite advancements in understanding and treating B-cell malignancies, research into SMZL lags behind. This is due to its rarity compared to other lymphomas, making it a lower research priority. The scarcity of cases also hinders large-scale studies, leading to combining MZL subtypes in recent trials of novel agents. Although we have made recent advancements in understanding SMZL’s deregulated pathways and molecular subtypes, actionable biomarkers and definitive genetic/transcriptional signatures for clinical use remain lacking. Diagnosing SMZL without splenic histology remains challenging, often relying on multiple factors that may not be readily available. This leads to variability in treatment and outcomes. Highlighting key knowledge gaps, we propose questions to better understand disease pathogenesis and suggest approaches to address them.

### What is the non-coding mutation landscape of the disease?

The research community is yet to generate the genome-wide data on coding and non-coding mutations in SMZL, from matched WGS of tumour and germline duos. The previous WGS study by Kiel et al. [55] did not include matched germ-line material from the SMZL patients, which is critical to generating a catalogue of the mutational landscape of any tumour, particularly when it comes to the non-coding space. This is because comparison to germline sequencing is required for adequate accuracy and sensitivity for the detection of high-confidence somatic variants. In contrast, in other B-cell tumours, such as CLL, larger cohorts with matched germline material have identified an expansive panel of genomic regions and genes targeted by non-coding mutations, such as *PAX5* [132] and *BACH2* [133]. Further insights into the key mutational signatures from paired WGS would be crucial in understanding the mechanisms involved in disease development and could potentially yield insights into malignant transformation. From a patient perspective, mutational signatures could provide insight into exposure to potential genotoxins helping to manage cancer risk, and might identify suitable treatment options for patient subgroups, such as poly- adenosine diphosphate (ADP)-ribose polymerase (PARP) or check-point inhibitors in patients with mutational signatures that suggest a deficiency in homologous recombination and mismatch repair, respectively.

### What drives clinical outcomes in poor-risk SMZL?

This review outlines several studies highlighting the molecular features of poor-risk SMZL. Taking these studies together, a key aggressive subset can be defined, harbouring distinct, partially overlapping clinico-biological characteristics. Immunogenetically, these cases are defined by a highly biased usage of the IGHV1-2*04 allele, with intermediate levels of SHM that appear to persist throughout tumour development. At the genomic level, these cases harbour del(7q), genome complexity, short telomeres and mutations within key regulators of marginal-zone development and NF-κΒ signaling (*NOTCH2* and *KLF2*). This group of cases also harbours high levels of global hypermethylation, including deregulation of the PCR2 complex, and evidence of elevated historical tumour proliferation with the capacity for future cellular growth. However, not all cases carry all these features, so it will be important to understand which has the greatest impact on disease biology and clinical outcome; these studies will require high-quality biological material for omics and functional analysis, from well-characterised patients with extensive follow-up, drawn from a large international consortium.

### Can we deconvolute tumour and normal cells in the SMZL TME?

In humans, the MZ is located at the interface between the red and white pulp, where macrophages and resident B-cells can rapidly respond to antigens in the circulating blood supply. How the development of SMZL in the spleen might deregulate normal interactions between different cell populations remains poorly studied. In other tumour types, single-cell sequencing (SCS) technology has revealed insight into cellular components, functions, and interactions by circumventing noise caused by genetic and phenotypic variations across individual samples. In SMZL, it could enable direct measurement of molecular characteristics in thousands or even millions of individual cells, facilitating the characterization of cell types and revealing insights into sub-clonal dynamics, gene regulatory networks, transcription dynamics, and proteomic profiles. The precise identification of sub-clones could be crucial for understanding shifts in gene expression and for gaining insights into the SMZL TME and inflammatory cell milieu, where preliminary studies implicate a high-risk proinflammatory TME [134]. The importance of the TME and its contribution to different disease sub-types is shown in the study by Bonfiglio et al. [59], showing an “immune-suppressive” class encompassing macrophage, cytokine, and T-cell signatures, and an “immune-silent” class dominated by B-cell signatures. The presence of an ‘immune-suppressive’ subgroup opens the possibility of checkpoint inhibitor treatment in these patients.

The analysis of IGHV1-2*04 tumours in the spleen could further confirm the presence of ongoing SHM by using VDJ sequencing, and documenting interactions with T-cells by using TCR single-cell tracking. This type of immune repertoire analysis should maximize the development and deployment of future immunotherapies. SCS information could be powerful in predicting tumour progression, and transformation and for predicting drug sensitivity or resistance. As tumour cells evolve under treatment pressure, spontaneous mutations can confer drug resistance to a subset of cells, where single-cell proteomic analysis could enable a more accurate correlation between gene mutations and protein expression, elucidating diverse clinically relevant mechanisms. The application of spatial biology technologies could provide further insight into the organization and cell-to-cell interactions between tumour, immune and stromal cells, as well as their distributions within the SMZL TME. Cellular confirmation will ultimately be required, and whilst these studies are also rare, due to the paucity of variable material for study, they have already shown impaired T-cell and myeloid cell function in a proportion of patients [135–137].

### When will these new molecular insights be translated for the benefit of patients?

The findings outlined in this review have significant clinical relevance and promise to enhance diagnosis and patient stratification in the future, provided that standardized testing protocols can be established. Achieving this goal presents challenges, necessitating the use of assays that capture the most clinically informative immunogenetic and (epi)genomic data. A single platform could be developed to perform: IGHV analysis, identification of gene mutations and CNAs linked to survival (del(7q), *KLF2*, *NOTCH2*, *TP53*, *TNFAIP3*), as well as those aiding in the differential diagnosis (e.g., *MYD88* for WM, *BRAF* for HCL, *NOTCH1* and *SF3B1* for CLL, *PTPRD* for NMZL, and *MAP2K1* for HCL-V), and assessment of proliferative history using DNA methylation data. As genomic knowledge expands, these platforms could be augmented with additional prognostically relevant and therapeutic target lesions, potentially facilitating treatment monitoring. While comprehensive multi-omics analysis, including epigenetic profiling, may offer the most precise patient identification for precision medicine, its adoption must be economically justified by selecting optimal treatment approaches. Regardless of the technical approach, each molecular marker must undergo rigorous validation across multiple cohorts, retrospective studies, and prospective trials to establish robust associations with disease outcomes. Analytical and clinical validation, followed by international harmonization, regulatory approval, and ongoing accreditation, are essential steps. Furthermore, guidelines for interpretation and reporting, along with appropriate training for clinical referral and patient communication, are imperative for effective implementation in clinical practice.

## Conclusions

Addressing the knowledge gaps in SMZL requires concerted efforts from the scientific community to prioritize research into this rare lymphoma subtype. Collaborative initiatives that leverage multi-institutional partnerships and integration of comprehensive omics approaches hold promise in advancing our understanding of SMZL biology and identifying novel therapeutic targets. Additionally, the development of both pre-clinical models and clinical trials specifically designed for patients with SMZL is essential for evaluating the efficacy of targeted therapies and refining risk stratification algorithms. This will be particularly important in SMZL, as the potential to employ molecular information to improve risk-adapted stratification and guide therapy choice has not, as yet, been realised. In conclusion, despite the remarkable progress in the field of B-cell malignancies, the lack of comprehensive study into SMZL highlights the need for increased research efforts and resource allocation toward understanding the biology, genetics, and optimal management of this rare lymphoma subtype. By addressing these knowledge gaps, we can improve outcomes for patients with SMZL and contribute to the broader understanding of lymphomagenesis and improve treatment regimens.

## Abbreviations

BCR: B-cell receptor
CBL-MZ: clonal B-cell lymphocytosis of marginal zone origin
CLL: chronic lymphocytic leukaemia
CNAs: copy number alterations
CpG: cytosine-guanine
DLBCL: diffuse large B-cell lymphoma
epiCMIT: epigenetically determined cumulative mitoses
HCL: hairy cell leukaemia
HCL-V: hairy cell leukaemia variant
High-M: high genome-wide promoter methylation subgroup
IGHV: immunoglobulin heavy variable
IL-1: interleukin-1
MCLs: mantle cell lymphomas
MDR: minimally deleted region
MZLs: marginal zone lymphomas
NF-κΒ: nuclear factor kappaB
PRC2: Polycomb repressor complex 2
SBLPN: splenic B-cell lymphoma/leukaemia with prominent pucleoli
SDRPL: splenic diffuse red pulp lymphoma
SHM: somatic hypermutation
SMZL: splenic marginal zone lymphoma
TLR: toll-like receptor
TME: tumour microenvironment
TNFRs: tumour necrosis factor receptors
TRAF: tumour necrosis factor-receptor associated factor
WGS: whole-genome sequencing
WHO: World Health Organization

## Declarations

### Copyright

© The Author(s) 2024.

## References

[1] Schmid C, Kirkham N, Diss T, Isaacson PG (1992). Splenic marginal zone cell lymphoma. Am J Surg Pathol..

[2] Swerdlow SH, Campo E, Pileri SA, Harris NL, Stein H, Siebert R (2016). The 2016 revision of the World Health Organization classification of lymphoid neoplasms. Blood..

[3] Alaggio R, Amador C, Anagnostopoulos I, Attygalle AD, Araujo IBdO, Berti E (2022). The 5th edition of the World Health Organization Classification of Haematolymphoid Tumours: Lymphoid Neoplasms. Leukemia..

[4] Coupland SE, Du M, Ferry JA, Jong Dd, Khoury JD, Leoncini L, WHO 5th Edition Classification Project (2024). The fifth edition of the WHO classification of mature B-cell neoplasms: open questions for research. J Pathol..

[5] Liu L, Wang H, Chen Y, Rustveld L, Liu G, Du XL (2013). Splenic marginal zone lymphoma: a population-based study on the 2001-2008 incidence and survival in the United States. Leuk Lymphoma..

[6] Swerdlow SH, Campo E, Harris NL, Jaffe ES, Pileri SA, Stein H (2008). WHO Classification of Tumours of Haematopoietic and Lymphoid Tissues.

[7] Arcaini L, Rossi D, Paulli M (2016). Splenic marginal zone lymphoma: from genetics to management. Blood..

[8] Chacón JI, Mollejo M, Muñoz E, Algara P, Mateo M, Lopez L (2002). Splenic marginal zone lymphoma: clinical characteristics and prognostic factors in a series of 60 patients. Blood..

[9] Donzel M, Baseggio L, Fontaine J, Pesce F, Ghesquières H, Bachy E (2021). New Insights into the Biology and Diagnosis of Splenic Marginal Zone Lymphomas. Curr Oncol..

[10] Pérez-Chacón G, Llobet D, Pardo C, Pindado J, Choi Y, Reed JC (2012). TNFR-associated factor 2 deficiency in B lymphocytes predisposes to chronic lymphocytic leukemia/small lymphocytic lymphoma in mice. J Immunol..

[11] Zhang S, Xuan Z, Zhang L, Lu J, Song P, Zheng S (2020). Splenic marginal zone lymphoma: a case report and literature review. World J Surg Oncol..

[12] Bracci PM, Benavente Y, Turner JJ, Paltiel O, Slager SL, Vajdic CM (2014). Medical history, lifestyle, family history, and occupational risk factors for marginal zone lymphoma: the InterLymph Non-Hodgkin Lymphoma Subtypes Project. J Natl Cancer Inst Monogr..

[13] Santos PD, Panero J, Nagore VP, Stanganelli C, Bezares RF, Slavutsky I (2015). Telomere shortening associated with increased genomic complexity in chronic lymphocytic leukemia. Tumour Biol..

[14] Xiong W, Lv R, Li H, Li Z, Wang H, Liu W (2017). Prevalence of hepatitis B and hepatitis C viral infections in various subtypes of B-cell non-Hodgkin lymphoma: confirmation of the association with splenic marginal zone lymphoma. Blood Cancer J..

[15] Arcaini L, Paulli M (2010). Splenic marginal zone lymphoma: hydra with many heads?. Haematologica.

[16] Matutes E, Oscier D, Montalban C, Berger F, Callet-Bauchu E, Dogan A (2008). Splenic marginal zone lymphoma proposals for a revision of diagnostic, staging and therapeutic criteria. Leukemia..

[17] Parker H, McIver-Brown NR, Davis ZA, Parry M, Rose-Zerilli MJJ, Xochelli A (2018). CBL-MZ is not a single biological entity: evidence from genomic analysis and prolonged clinical follow-up. Blood Adv..

[18] Zucca E, Arcaini L, Buske C, Johnson PW, Ponzoni M, Raderer M, ESMO Guidelines Committee (2020). Electronic address: clinicalguidelines@esmo. Ann Oncol..

[19] Thieblemont C, Felman P, Callet-Bauchu E, Traverse-Glehen A, Salles G, Berger F (2003). Splenic marginal-zone lymphoma: a distinct clinical and pathological entity. Lancet Oncol..

[20] Camacho FI, Mollejo M, Mateo MS, Algara P, Navas C, Hernández JM (2001). Progression to large B-cell lymphoma in splenic marginal zone lymphoma: a description of a series of 12 cases. Am J Surg Pathol..

[21] Bastidas-Mora G, Beà S, Navarro A, Gine E, Costa D, Delgado J (2022). Clinico-biological features and outcome of patients with splenic marginal zone lymphoma with histological transformation. Br J Haematol..

[22] Du Y, Wang Y, Li Q, Chang X, Shen K, Zhang H (2024). Transformation to diffuse large B-cell lymphoma and its impact on survival in patients with marginal zone lymphoma: A population-based study. Int J Cancer..

[23] Sun X, Li H, Yang Y, Wu Y, Kang K, Liu Q (2024). Transformation risk and associated survival outcome of marginal zone lymphoma: A nationwide study. Ann Hematol..

[24] Walewska R, Eyre TA, Barrington S, Brady J, Fields P, Iyengar S, BSH Committee (2024). Guideline for the diagnosis and management of marginal zone lymphomas: A British Society of Haematology Guideline. Br J Haematol..

[25] Iannitto E, Bellei M, Amorim S, Ferreri AJM, Marcheselli L, Cesaretti M (2018). Efficacy of bendamustine and rituximab in splenic marginal zone lymphoma: results from the phase II BRISMA/IELSG36 study. Br J Haematol..

[26] Srinivasan L, Sasaki Y, Calado DP, Zhang B, Paik JH, DePinho RA (2009). PI3 kinase signals BCR-dependent mature B cell survival. Cell..

[27] Vanhaesebroeck B, Ali K, Bilancio A, Geering B, Foukas LC (2005). Signalling by PI3K isoforms: insights from gene-targeted mice. Trends Biochem Sci..

[28] Clipson A, Wang M, Leval Ld, Ashton-Key M, Wotherspoon A, Vassiliou G (2015). KLF2 mutation is the most frequent somatic change in splenic marginal zone lymphoma and identifies a subset with distinct genotype. Leukemia..

[29] Parry M, Rose-Zerilli MJ, Ljungström V, Gibson J, Wang J, Walewska R (2015). Genetics and Prognostication in Splenic Marginal Zone Lymphoma: Revelations from Deep Sequencing. Clin Cancer Res..

[30] Bikos V, Darzentas N, Hadzidimitriou A, Davis Z, Hockley S, Traverse-Glehen A (2012). Over 30% of patients with splenic marginal zone lymphoma express the same immunoglobulin heavy variable gene: ontogenetic implications. Leukemia..

[31] Zaragoza-Infante L, Agathangelidis A, Papaioannou M, Chatzidimitriou A, Stamatopoulos K (2020). The B cell receptor in marginal zone lymphoma ontogeny and evolution. Annals of Lymphoma.

[32] Piva R, Deaglio S, Famà R, Buonincontri R, Scarfò I, Bruscaggin A (2015). The Krüppel-like factor 2 transcription factor gene is recurrently mutated in splenic marginal zone lymphoma. Leukemia..

[33] Bahler DW, Pindzola JA, Swerdlow SH (2002). Splenic marginal zone lymphomas appear to originate from different B cell types. Am J Pathol..

[34] Bikos V, Karypidou M, Stalika E, Baliakas P, Xochelli A, Sutton L (2016). An Immunogenetic Signature of Ongoing Antigen Interactions in Splenic Marginal Zone Lymphoma Expressing IGHV1-2*04 Receptors. Clin Cancer Res..

[35] Warsame AA, Aasheim H, Nustad K, Trøen G, Tierens A, Wang V (2011). Splenic marginal zone lymphoma with VH1-02 gene rearrangement expresses poly- and self-reactive antibodies with similar reactivity. Blood..

[36] Brisou G, Verney A, Wenner T, Baseggio L, Felman P, Callet-Bauchu E (2014). A restricted IGHV gene repertoire in splenic marginal zone lymphoma is associated with autoimmune disorders. Haematologica..

[37] Leeksma AC, Baliakas P, Moysiadis T, Puiggros A, Plevova K, Kevie-Kersemaekers AVd (2021). Genomic arrays identify high-risk chronic lymphocytic leukemia with genomic complexity: a multi-center study. Haematologica..

[38] Kujawski L, Ouillette P, Erba H, Saddler C, Jakubowiak A, Kaminski M (2008). Genomic complexity identifies patients with aggressive chronic lymphocytic leukemia. Blood..

[39] Salido M, Baró C, Oscier D, Stamatopoulos K, Dierlamm J, Matutes E (2010). Cytogenetic aberrations and their prognostic value in a series of 330 splenic marginal zone B-cell lymphomas: a multicenter study of the Splenic B-Cell Lymphoma Group. Blood..

[40] Andersen CL, Gruszka-Westwood A, Atkinson S, Matutes E, Catovsky D, Pedersen RK (2005). Recurrent genomic imbalances in B-cell splenic marginal-zone lymphoma revealed by comparative genomic hybridization. Cancer Genet Cytogenet..

[41] Dierlamm J, Pittaluga S, Wlodarska I, Stul M, Thomas J, Boogaerts M (1996). Marginal zone B-cell lymphomas of different sites share similar cytogenetic and morphologic features. Blood..

[42] Bagacean C, Tempescul A, Ternant D, Banet A, Douet-Guilbert N, Bordron A (2019). 17p deletion strongly influences rituximab elimination in chronic lymphocytic leukemia. J Immunother Cancer..

[43] Watkins AJ, Huang Y, Ye H, Chanudet E, Johnson N, Hamoudi R (2010). Splenic marginal zone lymphoma: characterization of 7q deletion and its value in diagnosis. J Pathol..

[44] Solé F, Salido M, Espinet B, Garcia JL, Climent JAM, Granada I (2001). Splenic marginal zone B-cell lymphomas: two cytogenetic subtypes, one with gain of 3q and the other with loss of 7q. Haematologica..

[45] Robledo C, García JL, Benito R, Flores T, Mollejo M, Martínez-Climent J (2011). Molecular characterization of the region 7q22.1 in splenic marginal zone lymphomas. PLoS One..

[46] Watkins AJ, Hamoudi RA, Zeng N, Yan Q, Huang Y, Liu H (2012). An integrated genomic and expression analysis of 7q deletion in splenic marginal zone lymphoma. PLoS One..

[47] Mateo M, Mollejo M, Villuendas R, Algara P, Sanchez-Beato M, Martínez P (1999). 7q31-32 allelic loss is a frequent finding in splenic marginal zone lymphoma. Am J Pathol..

[48] Rinaldi A, Mian M, Chigrinova E, Arcaini L, Bhagat G, Novak U (2011). Genome-wide DNA profiling of marginal zone lymphomas identifies subtype-specific lesions with an impact on the clinical outcome. Blood..

[49] McKeithan TW, Rowley JD, Shows TB, Diaz MO (1987). Cloning of the chromosome translocation breakpoint junction of the t(14;19) in chronic lymphocytic leukemia. Proc Natl Acad Sci U S A..

[50] Küppers R (2024). Distinct t(14;19) translocation patterns in atypical chronic lymphocytic leukemia and marginal zone lymphomas. Haematologica..

[51] Grau M, López C, Navarro A, Frigola G, Nadeu F, Clot G (2023). Unraveling the genetics of transformed splenic marginal zone lymphoma. Blood Adv..

[52] Fresquet V, Robles EF, Parker A, Martinez-Useros J, Mena M, Malumbres R (2012). High-throughput sequencing analysis of the chromosome 7q32 deletion reveals IRF5 as a potential tumour suppressor in splenic marginal-zone lymphoma. Br J Haematol..

[53] Parry M, Rose-Zerilli MJJ, Gibson J, Ennis S, Walewska R, Forster J (2013). Whole exome sequencing identifies novel recurrently mutated genes in patients with splenic marginal zone lymphoma. PLoS One..

[54] Rossi D, Trifonov V, Fangazio M, Bruscaggin A, Rasi S, Spina V (2012). The coding genome of splenic marginal zone lymphoma: activation of NOTCH2 and other pathways regulating marginal zone development. J Exp Med..

[55] Kiel MJ, Velusamy T, Betz BL, Zhao L, Weigelin HG, Chiang MY (2012). Whole-genome sequencing identifies recurrent somatic *NOTCH2* mutations in splenic marginal zone lymphoma. J Exp Med..

[56] Campos-Martín Y, Martínez N, Martínez-López A, Cereceda L, Casado F, Algara P (2017). Clinical and diagnostic relevance of *NOTCH2*-and *KLF2*-mutations in splenic marginal zone lymphoma. Haematologica..

[57] Lechner M, Engleitner T, Babushku T, Schmidt-Supprian M, Rad R, Strobl LJ (2021). Notch2-mediated plasticity between marginal zone and follicular B cells. Nat Commun..

[58] Saito T, Chiba S, Ichikawa M, Kunisato A, Asai T, Shimizu K (2003). Notch2 is preferentially expressed in mature B cells and indispensable for marginal zone B lineage development. Immunity..

[59] Bonfiglio F, Bruscaggin A, Guidetti F, Bergamo LTd, Faderl M, Spina V (2022). Genetic and phenotypic attributes of splenic marginal zone lymphoma. Blood..

[60] Rosati E, Baldoni S, Falco FD, Papa BD, Dorillo E, Rompietti C (2018). NOTCH1 Aberrations in Chronic Lymphocytic Leukemia. Front Oncol..

[61] Martinez D, Navarro A, Martinez-Trillos A, Molina-Urra R, Gonzalez-Farre B, Salaverria I (2016). NOTCH1, TP53, and MAP2K1 Mutations in Splenic Diffuse Red Pulp Small B-cell Lymphoma Are Associated With Progressive Disease. Am J Surg Pathol..

[62] Silkenstedt E, Arenas F, Colom-Sanmartí B, Xargay-Torrent S, Higashi M, Giró A (2019). Notch1 signaling in NOTCH1-mutated mantle cell lymphoma depends on Delta-Like ligand 4 and is a potential target for specific antibody therapy. J Exp Clin Cancer Res..

[63] Kuroda K, Han H, Tani S, Tanigaki K, Tun T, Furukawa T (2003). Regulation of marginal zone B cell development by MINT, a suppressor of Notch/RBP-J signaling pathway. Immunity..

[64] Honma K, Tsuzuki S, Nakagawa M, Tagawa H, Nakamura S, Morishima Y (2009). TNFAIP3/A20 functions as a novel tumor suppressor gene in several subtypes of non-Hodgkin lymphomas. Blood..

[65] Turpaev KT (2020). Transcription Factor KLF2 and Its Role in the Regulation of Inflammatory Processes. Biochemistry (Mosc)..

[66] Hoek KL, Gordy LE, Collins PL, Parekh VV, Aune TM, Joyce S (2010). Follicular B cell trafficking within the spleen actively restricts humoral immune responses. Immunity..

[67] Winkelmann R, Sandrock L, Kirberg J, Jäck H, Schuh W (2014). KLF2–a negative regulator of pre-B cell clonal expansion and B cell activation. PLoS One..

[68] Hart GT, Wang X, Hogquist KA, Jameson SC (2011). Krüppel-like factor 2 (KLF2) regulates B-cell reactivity, subset differentiation, and trafficking molecule expression. Proc Natl Acad Sci U S A..

[69] Yan Q, Huang Y, Watkins AJ, Kocialkowski S, Zeng N, Hamoudi RA (2012). BCR and TLR signaling pathways are recurrently targeted by genetic changes in splenic marginal zone lymphomas. Haematologica..

[70] Moore CR, Liu Y, Shao C, Covey LR, Morse HC 3rd, Xie P (2012). Specific deletion of TRAF3 in B lymphocytes leads to B-lymphoma development in mice. Leukemia..

[71] Aubrey BJ, Strasser A, Kelly GL (2016). Tumor-Suppressor Functions of the TP53 Pathway. Cold Spring Harb Perspect Med..

[72] de Groen RAL, Schrader AMR, Kersten MJ, Pals ST, Vermaat JSP (2019). MYD88 in the driver's seat of B-cell lymphomagenesis: from molecular mechanisms to clinical implications. Haematologica..

[73] Martinez-Lopez A, Curiel-Olmo S, Mollejo M, Cereceda L, Martinez N, Montes-Moreno S (2015). MYD88 (L265P) somatic mutation in marginal zone B-cell lymphoma. Am J Surg Pathol..

[74] Wang S, Charbonnier L, Rivas MN, Georgiev P, Li N, Gerber G (2015). MyD88 Adaptor-Dependent Microbial Sensing by Regulatory T Cells Promotes Mucosal Tolerance and Enforces Commensalism. Immunity..

[75] Yu X, Li W, Deng Q, Li L, Hsi ED, Young KH (2018). *MYD88* L265P Mutation in Lymphoid Malignancies. Cancer Res..

[76] Varettoni M, Arcaini L, Zibellini S, Boveri E, Rattotti S, Riboni R (2013). Prevalence and clinical significance of the MYD88 (L265P) somatic mutation in Waldenstrom's macroglobulinemia and related lymphoid neoplasms. Blood..

[77] Alcoceba M, García-Álvarez M, Medina A, Maldonado R, González-Calle V, Chillón MC (2022). *MYD88* Mutations: Transforming the Landscape of IgM Monoclonal Gammopathies. Int J Mol Sci..

[78] Rovira J, Karube K, Valera A, Colomer D, Enjuanes A, Colomo L (2016). MYD88 L265P Mutations, But No Other Variants, Identify a Subpopulation of DLBCL Patients of Activated B-cell Origin, Extranodal Involvement, and Poor Outcome. Clin Cancer Res..

[79] Weller S, Bonnet M, Delagreverie H, Israel L, Chrabieh M, Maródi L (2012). IgM^+^IgD^+^CD27^+^ B cells are markedly reduced in IRAK-4-, MyD88-, and TIRAP- but not UNC-93B-deficient patients. Blood..

[80] Zhang J, Dominguez-Sola D, Hussein S, Lee J, Holmes AB, Bansal M (2015). Disruption of KMT2D perturbs germinal center B cell development and promotes lymphomagenesis. Nat Med..

[81] Ortega-Molina A, Boss IW, Canela A, Pan H, Jiang Y, Zhao C (2015). The histone lysine methyltransferase KMT2D sustains a gene expression program that represses B cell lymphoma development. Nat Med..

[82] Mondello P, Tadros S, Teater M, Fontan L, Chang AY, Jain N (2020). Selective Inhibition of HDAC3 Targets Synthetic Vulnerabilities and Activates Immune Surveillance in Lymphoma. Cancer Discov..

[83] Zhang J, Vlasevska S, Wells VA, Nataraj S, Holmes AB, Duval R (2017). The CREBBP Acetyltransferase Is a Haploinsufficient Tumor Suppressor in B-cell Lymphoma. Cancer Discov..

[84] Hernández JM, García JL, Gutiérrez NC, Mollejo M, Martínez-Climent JA, Flores T (2001). Novel genomic imbalances in B-cell splenic marginal zone lymphomas revealed by comparative genomic hybridization and cytogenetics. Am J Pathol..

[85] Remstein ED, Law M, Mollejo M, Piris MA, Kurtin PJ, Dogan A (2008). The prevalence of IG translocations and 7q32 deletions in splenic marginal zone lymphoma. Leukemia..

[86] Carbo-Meix A, Guijarro F, Wang L, Grau M, Royo R, Frigola G (2024). *BCL3* rearrangements in B-cell lymphoid neoplasms occur in two breakpoint clusters associated with different diseases. Haematologica..

[87] Sasaki Y, Iwai K (2016). Roles of the NF-κB Pathway in B-Lymphocyte Biology. Curr Top Microbiol Immunol..

[88] Liu T, Zhang L, Joo D, Sun S (2017). NF-κB signaling in inflammation. Signal Transduct Target Ther..

[89] Shembade N, Harhaj EW (2012). Regulation of NF-κB signaling by the A20 deubiquitinase. Cell Mol Immunol..

[90] Sasaki Y, Derudder E, Hobeika E, Pelanda R, Reth M, Rajewsky K (2006). Canonical NF-kappaB activity, dispensable for B cell development, replaces BAFF-receptor signals and promotes B cell proliferation upon activation. Immunity..

[91] Nagel D, Vincendeau M, Eitelhuber AC, Krappmann D (2014). Mechanisms and consequences of constitutive NF-κB activation in B-cell lymphoid malignancies. Oncogene..

[92] Rossi D, Deaglio S, Dominguez-Sola D, Rasi S, Vaisitti T, Agostinelli C (2011). Alteration of *BIRC3* and multiple other NF-κB pathway genes in splenic marginal zone lymphoma. Blood..

[93] Sakakibara S, Espigol-Frigole G, Gasperini P, Uldrick TS, Yarchoan R, Tosato G (2013). A20/TNFAIP3 inhibits NF-κB activation induced by the Kaposi’s sarcoma-associated herpesvirus vFLIP oncoprotein. Oncogene..

[94] Song HY, Rothe M, Goeddel DV (1996). The tumor necrosis factor-inducible zinc finger protein A20 interacts with TRAF1/TRAF2 and inhibits NF-kappaB activation. Proc Natl Acad Sci U S A..

[95] Zhu S, Jin J, Gokhale S, Lu AM, Shan H, Feng J (2018). Genetic Alterations of TRAF Proteins in Human Cancers. Front Immunol..

[96] Rahal R, Frick M, Romero R, Korn JM, Kridel R, Chan FC (2014). Pharmacological and genomic profiling identifies NF-κB-targeted treatment strategies for mantle cell lymphoma. Nat Med..

[97] Lenz G, Wright GW, Emre NCT, Kohlhammer H, Dave SS, Davis RE (2008). Molecular subtypes of diffuse large B-cell lymphoma arise by distinct genetic pathways. Proc Natl Acad Sci U S A..

[98] Fonte E, Agathangelidis A, Reverberi D, Ntoufa S, Scarfò L, Ranghetti P (2015). Toll-like receptor stimulation in splenic marginal zone lymphoma can modulate cell signaling, activation and proliferation. Haematologica..

[99] Ngo VN, Young RM, Schmitz R, Jhavar S, Xiao W, Lim K (2011). Oncogenically active MYD88 mutations in human lymphoma. Nature..

[100] Das H, Kumar A, Lin Z, Patino WD, Hwang PM, Feinberg MW (2006). Kruppel-like factor 2 (KLF2) regulates proinflammatory activation of monocytes. Proc Natl Acad Sci U S A..

[101] Alderuccio JP, Lossos IS (2022). NOTCH signaling in the pathogenesis of splenic marginal zone lymphoma-opportunities for therapy. Leuk Lymphoma..

[102] Garis M, Garrett-Sinha LA (2021). Notch Signaling in B Cell Immune Responses. Front Immunol..

[103] Kopan R (2012). Notch signaling. Cold Spring Harb Perspect Biol..

[104] Karube K, Enjuanes A, Dlouhy I, Jares P, Martin-Garcia D, Nadeu F (2018). Integrating genomic alterations in diffuse large B-cell lymphoma identifies new relevant pathways and potential therapeutic targets. Leukemia..

[105] Wang NJ, Sanborn Z, Arnett KL, Bayston LJ, Liao W, Proby CM (2011). Loss-of-function mutations in Notch receptors in cutaneous and lung squamous cell carcinoma. Proc Natl Acad Sci U S A..

[106] Shanmugam V, Craig JW, Hilton LK, Nguyen MH, Rushton CK, Fahimdanesh K (2021). Notch activation is pervasive in SMZL and uncommon in DLBCL: implications for Notch signaling in B-cell tumors. Blood Adv..

[107] Oquendo CJ, Parker H, Oscier D, Ennis S, Gibson J, Strefford JC (2019). Systematic Review of Somatic Mutations in Splenic Marginal Zone Lymphoma. Sci Rep..

[108] Gailllard B, Cornillet-Lefebvre P, Le Q, Maloum K, Pannetier M, Lecoq-Lafon C (2021). Clinical and biological features of B-cell neoplasms with CDK6 translocations: an association with a subgroup of splenic marginal zone lymphomas displaying frequent CD5 expression, prolymphocytic cells, and TP53 abnormalities. Br J Haematol..

[109] Spina V, Rossi D (2017). Molecular pathogenesis of splenic and nodal marginal zone lymphoma. Best Pract Res Clin Haematol..

[110] Kelso TWR, Porter DK, Amaral ML, Shokhirev MN, Benner C, Hargreaves DC (2017). Chromatin accessibility underlies synthetic lethality of SWI/SNF subunits in ARID1A-mutant cancers. Elife..

[111] Lakshminarasimhan R, Andreu-Vieyra C, Lawrenson K, Duymich CE, Gayther SA, Liang G (2017). Down-regulation of ARID1A is sufficient to initiate neoplastic transformation along with epigenetic reprogramming in non-tumorigenic endometriotic cells. Cancer Lett..

[112] Oakes CC, Martin-Subero JI (2018). Insight into origins, mechanisms, and utility of DNA methylation in B-cell malignancies. Blood..

[113] Oakes CC, Seifert M, Assenov Y, Gu L, Przekopowitz M, Ruppert AS (2016). DNA methylation dynamics during B cell maturation underlie a continuum of disease phenotypes in chronic lymphocytic leukemia. Nat Genet..

[114] Arribas AJ, Bertoni F (2017). Methylation patterns in marginal zone lymphoma. Best Pract Res Clin Haematol..

[115] Arribas AJ, Rinaldi A, Mensah AA, Kwee I, Cascione L, Robles EF (2015). DNA methylation profiling identifies two splenic marginal zone lymphoma subgroups with different clinical and genetic features. Blood..

[116] Duran-Ferrer M, Clot G, Nadeu F, Beekman R, Baumann T, Nordlund J (2020). The proliferative history shapes the DNA methylome of B-cell tumors and predicts clinical outcome. Nat Cancer..

[117] Parker H, Mirandari A, Oquendo CJ, Duran-Ferrer M, Stevens B, Buermann L Proliferative History Is a Novel Driver of Clinical Outcome in Splenic Marginal Zone Lymphoma. https://www.medrxiv.org/content/10.1101/2024.01.16.24301320v1.

[118] Ruiz-Ballesteros E, Mollejo M, Rodriguez A, Camacho FI, Algara P, Martinez N (2005). Splenic marginal zone lymphoma: proposal of new diagnostic and prognostic markers identified after tissue and cDNA microarray analysis. Blood..

[119] Trøen G, Nygaard V, Jenssen T, Ikonomou IM, Tierens A, Matutes E (2004). Constitutive expression of the AP-1 transcription factors c-jun, junD, junB, and c-fos and the marginal zone B-cell transcription factor Notch2 in splenic marginal zone lymphoma. J Mol Diagn..

[120] Robinson JE, Greiner TC, Bouska AC, Iqbal J, Cutucache CE (2020). Identification of a Splenic Marginal Zone Lymphoma Signature: Preliminary Findings With Diagnostic Potential. Front Oncol..

[121] Calin GA, Dumitru CD, Shimizu M, Bichi R, Zupo S, Noch E (2002). Frequent deletions and down-regulation of micro- RNA genes miR15 and miR16 at 13q14 in chronic lymphocytic leukemia. Proc Natl Acad Sci U S A..

[122] Klein U, Lia M, Crespo M, Siegel R, Shen Q, Mo T (2010). The DLEU2/miR-15a/16-1 cluster controls B cell proliferation and its deletion leads to chronic lymphocytic leukemia. Cancer Cell..

[123] Ruiz-Ballesteros E, Mollejo M, Mateo M, Algara P, Martínez P, Piris MA (2007). MicroRNA losses in the frequently deleted region of 7q in SMZL. Leukemia..

[124] Arribas AJ, Gómez-Abad C, Sánchez-Beato M, Martinez N, Dilisio L, Casado F (2013). Splenic marginal zone lymphoma: comprehensive analysis of gene expression and miRNA profiling. Mod Pathol..

[125] Peveling-Oberhag J, Crisman G, Schmidt A, Döring C, Lucioni M, Arcaini L (2012). Dysregulation of global microRNA expression in splenic marginal zone lymphoma and influence of chronic hepatitis C virus infection. Leukemia..

[126] Scarola M, Schoeftner S, Schneider C, Benetti R (2010). miR-335 directly targets Rb1 (pRb/p105) in a proximal connection to p53-dependent stress response. Cancer Res..

[127] Karaayvaz M, Zhai H, Ju J (2013). miR-129 promotes apoptosis and enhances chemosensitivity to 5-fluorouracil in colorectal cancer. Cell Death Dis..

[128] Yamaguchi K, Abdelbaky S, Arons E, Cross M, Wu YZ, Weigel C (2023). DNA Methylation-Based Classification of Hairy Cell Leukemia and Splenic B Cell Lymphoma. Blood.

[129] Hopper MA, Wenzl K, Hartert KT, Krull JE, Dropik AR, Novak JP (2023). Molecular classification and identification of an aggressive signature in low-grade B-cell lymphomas. Hematol Oncol..

[130] Calado DP, Zhang B, Srinivasan L, Sasaki Y, Seagal J, Unitt C (2010). Constitutive canonical NF-κB activation cooperates with disruption of BLIMP1 in the pathogenesis of activated B cell-like diffuse large cell lymphoma. Cancer Cell..

[131] Davies AJ, Rosenwald A, Wright G, Lee A, Last KW, Weisenburger DD (2007). Transformation of follicular lymphoma to diffuse large B-cell lymphoma proceeds by distinct oncogenic mechanisms. Br J Haematol..

[132] Puente XS, Beà S, Valdés-Mas R, Villamor N, Gutiérrez-Abril J, Martín-Subero JI (2015). Non-coding recurrent mutations in chronic lymphocytic leukaemia. Nature..

[133] Robbe P, Ridout KE, Vavoulis DV, Dréau H, Kinnersley B, Denny N (2022). Whole-genome sequencing of chronic lymphocytic leukemia identifies subgroups with distinct biological and clinical features. Nat Genet..

[134] Franco G, Guarnotta C, Frossi B, Piccaluga PP, Boveri E, Gulino A (2014). Bone marrow stroma CD40 expression correlates with inflammatory mast cell infiltration and disease progression in splenic marginal zone lymphoma. Blood..

[135] Anagnostou T, Yang Z, Jalali S, Kim HJ, Larson DP, Tang X (2023). Characterization of immune exhaustion and suppression in the tumor microenvironment of splenic marginal zone lymphoma. Leukemia..

[136] Tang X, Yang Z, Kim HJ, Anagnostou T, Yu Y, Wu X (2022). Phenotype, Function, and Clinical Significance of CD26+ and CD161+Tregs in Splenic Marginal Zone Lymphoma. Clin Cancer Res..

[137] Vincent-Fabert C, Soubeyran I, Velasco V, Parrens M, Jeannet R, Lereclus E (2019). Inflamed phenotype of splenic marginal zone B-cell lymphomas with expression of PD-L1 by intratumoral monocytes/macrophages and dendritic cells. Cell Mol Immunol..

